# A Novel Mutation in the *NCF2* Gene in a CGD Patient With Chronic Recurrent Pneumopathy

**DOI:** 10.3389/fped.2019.00391

**Published:** 2019-09-27

**Authors:** Jose Antonio Tavares de Albuquerque, Alessandra Miramontes Lima, Edgar Borges de Oliveira Junior, Edson Kiyotaka Ishizuka, Walmir Cutrim Aragão-Filho, Nuria Bengala Zurro, Sônia Mayumi Chiba, Fátima Rodrigues Fernandes, Antonio Condino-Neto

**Affiliations:** ^1^Immunogenic Inc, São Paulo, Brazil; ^2^PENSI Institute - Jose Luiz Egydio Setubal Foundation, Sabará Hospital, São Paulo, Brazil; ^3^Department of Immunology, Institute of Biomedical Sciences, University of São Paulo, São Paulo, Brazil; ^4^Sabará Hospital, São Paulo, Brazil; ^5^Department of Pediatrics, Federal University of São Paulo, São Paulo, Brazil

**Keywords:** chronic granulomatous disease, NADPH oxidase, *NCF2* gene, novel mutation, interferon-gamma

## Abstract

Chronic granulomatous disease (CGD) is an inherited, genetically heterogeneous disease characterized by defective phagocytic cell microbicidal function, leading to increased susceptibility to bacterial and fungal infections. CGD is caused by mutations in components of the nicotinamide adenine dinucleotide phosphate (NADPH) oxidase system, which is responsible for reactive oxygen species production during phagocytosis. Mutations in the neutrophil cytosolic factor 2 (*NCF2*) gene account for <5% of all cases. Here, we report a case of a 2-year-old female with persistent recurrent pneumopathy, even under trimethoprim-sulfamethoxazole (TMP-SMX) and itraconazole prophylaxis combined with IFNγ treatment. Genetic analysis revealed a novel homozygous mutation in *NCF2*, sequence depletion in a splicing region (c.256_257+2delAAGT NM_000433), leading to a K86Ifs^*^2 residue change in the p67^−phox^ protein.

## Introduction

Chronic granulomatous disease (CGD) is a severe immunodeficiency syndrome caused by functional impairment of the nicotinamide adenine dinucleotide phosphate (NADPH) oxidase complex in neutrophilic granulocytes and monocytes. Patients with CGD are generally diagnosed in early childhood after developing recurrent and severe infections, dysregulated granulomatous inflammation, or autoimmunity (1). Mutations in NADPH oxidase affect the generation of reactive oxygen species (ROS) within phagosomes and is essential for the normal killing of bacteria and fungi (2).

Subunits of NADPH oxidase are located at the membrane and in the cytoplasm. For example, the catalytic glycoprotein gp91^−phox^ and the non-glycosylated protein p22^−phox^ are located at the cell membrane and together form the heterodimer cytochrome *b*_558_. Upon induction, the cytosolic proteins p47^−phox^, p67^−phox^, and p40^−phox^ migrate toward cytochrome *b*_558_ to activate the enzyme complex and produce ROS (1, 2).

Mutations in all five structural genes of NADPH oxidase have been implicated in CGD, affecting 1 in 250,000 individuals. In addition, the X-linked CGD caused by cytochrome b-245 beta chain (*CYBB*) gene mutations is the most common form affecting gp91^−phox^ and accounts for 74% of all CGD patients in Latin America (3). Defects in the neutrophil cytosolic factor 1 (*NCF1*) gene encoding the p47^−phox^ subunit represent the most common form of autosomal recessive (AR) CGD, accounting for 20% of all cases. In contrast, mutations in the genes encoding p22^−phox^ (cytochrome b-245 alpha chain, *CYBA*) and p67^−phox^ (neutrophil cytosolic factor 2, *NCF2*) are rare and account for <5% of all cases of CGD (1, 4).

As a result of the antimicrobial activity defect in CGD, these patients are susceptible to infections such as pneumonia, lymphadenitis, cutaneous and hepatic abscesses, osteomyelitis and, septicemia (3, 5). Pneumonia is the most common pulmonary infection, and patients may also have complications such as lung abscesses, empyema, and hilar lymphadenopathy.

The microorganisms implicated in CGD patient infections include *Staphylococcus aureus, Aspergillus* spp., the *Burkholderia cepacia* complex, *Candida* spp., enteric gram-negative bacteria, *Mycobacterium tuberculosis*, and *Serratia marcescens* (3, 5). Overall, treatment with lifelong antibiotic and antifungal prophylaxis and interferon-gamma (IFNγ) have a clear benefit, with a reduction in both the number and severity of infections in most CGD patients. Regardless, the only curative option is allogenic hematopoietic stem cell transplantation (HSCT), which shows very promising results in well-defined circumstances (6, 7).

Here, we describe a case of autosomal recessive CGD (AR-CGD) diagnosed in a 3-year-old female. The patient carried a novel mutation in *NCF2* leading to a K86Ifs^*^2 residue change in the p67^−phox^ protein. She presented with recurrent pneumonia, even under trimethoprim-sulfamethoxazole (TMP-SMX) and itraconazole prophylaxis plus IFNγ therapy.

## Ethical Approval

This study was approved by the Ethics and Research Committees in Humans of the Sabará Hospital and Institute of Biomedical Sciences, University of São Paulo, in accordance with the Declaration of Helsinki. The patient's parents provided written informed consent before the investigation.

## Case Report

The patient was a 3-year-old girl, the only child of Lebanese parents without direct consanguinity, with early onset of severe, recurrent pulmonary infections. Her first clinical manifestation occurred at 3 months of life, when she presented with fever without localizing signs. At that time, she was treated with intramuscular ceftriaxone for 3 days until she was admitted at an Intensive Care Unit due to bilateral pneumonia and pleural effusion. Then, she was submitted to pleural drainage and treatment with polymyxin, meropenem, vancomycin, and linezolid for 14 days. No etiological agents were identified in blood cultures and pleural fluids. At discharge, cefuroxime was prescribed for 10 days due to the persistent infection that was identified by radiological images.

**Figure 1:**
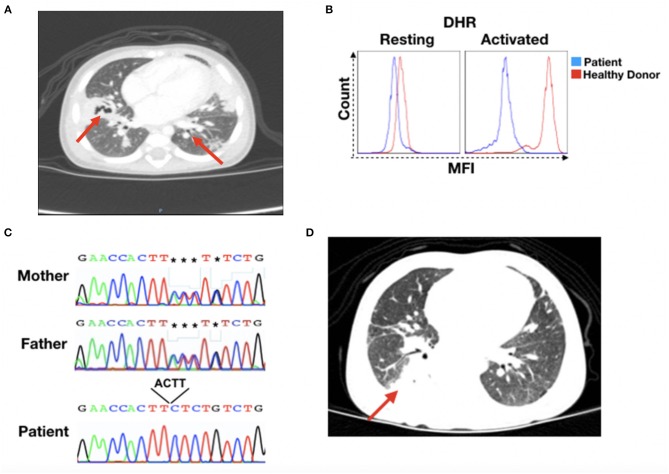
CGD patient's clinical recordings. **(A)** Patient's chest X-ray at 15 months of age. Red arrows indicate lesion sites. **(B)** Dihydrorhodamine (DHR) for patient and healthy donor granulocytes in the resting or activated state. **(C)** DNA sequencing results for the *NCF2* gene. Chromatographs for the mother (top), father (middle), and patient (bottom). The black box shows the sequence depletion in a splicing region (c.256_257+2delAAGT NM_000433), resulting in a K86Ifs*2 residue change in the p67^−phox^ protein. The reverse nucleotide sequences are shown. **(D)** Patient's thorax computed tomography at 17 months of age. The red arrow indicates the lesion site. MFI, mean fluorescence intensity.

At 4 months of age, she presented with oral moniliasis and Bacillus Calmette-Guérin (BCG) scar suppuration with slow healing. She was hospitalized for the second time at 6 months of age when she was diagnosed with bronchiolitis, with identification of *Haemophilus parainfluenzae* biotype I. She developed bilateral pneumonia, which did not respond adequately to ceftriaxone treatment and evolved to necrotizing pneumonia (Figure 1A). At that time, the patient was transferred to our hospital for evaluation by pulmonologists and immunologists. The patient was diagnosed with necrotizing pneumonia; left lung abscess; anemia, most likely secondary to infection; and stomatitis. She received a transfusion of red blood cell concentrate and was treated with ceftriaxone and linezolid and subsequently treated with meropenem and amphotericin B. Culture of material collected from bronchoscopy and bronchoalveolar lavage showed no growth of aerobic or anaerobic bacteria.

At this time, the patient was submitted to an immunological investigation, which revealed normal immunoglobulin levels, lymphocyte subset numbers, and complement system. Nevertheless, a dihydrorhodamine (DHR) test showed that the patient's stimulated granulocytes presented abnormal ROS production, a characteristic of CGD patients (Figure 1B). Thus, CGD prophylaxis was initiated with the administration of Bactrim, itraconazole, and IFNγ.

Despite prophylactic treatment, the patient continued to need hospitalization for pneumonia at 8 and 11 months of age that required systemic antibiotics. The first genetic analysis was performed on one of these occasions, although Sanger sequencing revealed no mutations in the *NCF1* gene (data not shown). At this time, the patient's mother reported that one of the patient's female cousin had received two transplants for an unidentified immunological problem.

At 23 months of age, the patient presented with adenitis and cervical abscess, which were treated with ceftriaxone, clindamycin, and surgical drainage. She was discharged with cefuroxima treatment; however, 1 week later, she required medical assistance after developing right lobe pneumonia and atelectasis. These last conditions were treated with cefepime and prednisolone.

Whole-exome sequencing was performed to confirm or discard a CGD diagnosis, and sequence analysis revealed a mutation (c.256_257+2delAAGT NM_000433) in the *NCF2* gene that leads to a K86Ifs^*^2 residue change in the p67^−phox^ protein, which was confirmed by Sanger sequencing (Figure 1C). Sanger analysis also showed that the parents were heterozygous for the mutation. The sequence data were analyzed using the National Center for Biotechnology Information (NCBI) (www.ncbi.nlm.nih.gov) and Ensembl SNP (www.ensembl.org) databases, confirming this novel mutation in *NCF2*.

Prophylactic medications were maintained during patient follow-up. However, at 2 years and 7 months of age, she was readmitted with pneumonia that was treated with ceftazidima. Thorax computed tomography revealed pulmonary consolidation in the upper segment of the right lower lobe and areas of retractable stretch marks, with a bilateral sparse sequential aspect that was more evident in the posterior segment of the right upper lobe, the middle lobe, and the lingula (Figure 1D). Bronchoscopy showed non-purulent laryngotracheobronchitis, with negative results for acid-alcohol resistant bacilli, fungi, cytomegalovirus or *Pneumocystis jirovecii*, as well as normal serum galactomannan levels.

At 3 years and 5 months of age, the child was hospitalized with jugal abscess arising from a dental procedure. The abscess was drained, and she received ceftriaxone, clindamycin and oral steroids for 5 days. Once she presented an anaphylactic reaction to ceftriaxone, the patient received epinephrine and continued treatment with clindamycin and steroids for an additional 3 days. Thereafter, she was discharged under treatment with sulfamethoxazole and trimethoprim.

## Discussion

CGD comprises a group of genetic disorders of the NADPH oxidase complex of phagocytes that results in defective microbicidal activity due to dysregulated ROS generation. Patients with CGD suffer from recurrent life-threatening bacterial and fungal infections and from uncontrolled inflammatory responses leading to granuloma formation in multiple organs. These infections are caused by catalase-positive microorganisms, *Staphylococcus* spp., *Serratia* spp., *Aspergillus* spp., and *Burkholderia cepacia*, as well as by BCG and *M. tuberculosis* in developing countries (1, 3, 5).

Here, we report the case of a patient who developed recurrent pneumonia beginning at 3 months of age. At this age, she was admitted to an intensive care unit due to bilateral pneumonia and pleural effusion. At 6 months of age, she developed bilateral pneumonia that evolved to necrotizing pneumonia, presenting with left lung abscess, anemia, and stomatitis. These clinical manifestations are present in CGD patients in which the lungs, followed by the gastrointestinal tract, lymph nodes, and skin are the most prevalent infection sites. Recurrent pneumonia is the most frequent clinical condition associated with CGD and may be complicated by abscess formation or pleural effusion (1, 3, 5, 8). Moreover, respiratory complications are among the signs and symptoms that constitute significant causes of mortality and morbidity in CGD.

In a retrospective analysis involving 71 patients with CGD and mycobacterial infection (9), 75% of these patients (53 individuals) presented adverse effects from BCG vaccination. This finding brings up the necessity of a CGD investigation when such adverse effects are observed. Considering the occurrence of BCG scar suppuration with slow healing when our patient was 4 months old and the characteristics of her infections with evolution to empyema and pulmonary abscess, we investigated the hypothesis for primary immunodeficiency in phagocytes. Then, a DHR test was performed, and the results were suggestive of CGD.

Genetic investigations confirmed that the patient has AR-CGD due to a novel homozygous mutation in the *NCF2* gene. This mutation leads to sequence depletion in a splicing region (c.256_257+2delAAGT NM_000433), resulting in a K86Ifs^*^2 residue change in the p67^−phox^ protein. The occurrence of AR forms of CGD is well-correlated with the high frequency of consanguineous marriages in Western Asian countries, which includes the Lebanese Republic, the birth place of our patient. Indeed, in these countries, AR-CGD is more common (10–13) than the X-linked CGD form, which predominates in the United States (14), Europe (15–18), and Japan (19) (60% of cases). Nevertheless, our patient had Lebanese parents without direct consanguinity, and Sanger analysis showed that they were heterozygous for the same *NCF2* mutation probably inherited from a progenitor many generations ago.

The current prophylactic therapy for CGD is daily doses of TMP-SMX and itraconazole, which results in reduced bacterial and fungal infections among CGD patients. Indeed, lifelong prophylaxis with TMP-SMX (5 mg/kg/d up to 320 mg trimethoprim a day) has been used routinely since the 1970s, and it has reduced the incidence of serious non-fungal infections from 7.1 to 2.4 per 100 patient-months in patients with AR-CGD (20, 21). Moreover, prophylaxis with itraconazole (5 mg/kg/d up to 200 mg daily) can decrease fungal infection mortality and morbidity (22). The addition of IFNγ to CGD prophylactic therapy is still under debate, and this decision should be made on a case-by-case basis. For example, an Italian study did not find evidence of increased protection from infections in CGD patients treated with IFNγ (16). By contrast, another report showed that CGD patients treated with IFNγ (50 μg/m^2^ subcutaneously three times weekly) exhibited a clear reduction in severe infections without exacerbation of granulomatous or inflammatory complications in 67% of patients (23).

Prophylactic therapy is effective for all genetic types of CGD, and children younger than 10 years old appear to benefit the most. The use of IFNγ in CGD prophylaxis is particularly encouraged for patients experiencing increased infection frequency but should be interrupted in cases of adverse events, such as fever, fatigue, headache, rash, myalgia, abdominal pain, and/or granulomatous colitis (6, 7). Since our patient still presents persistent respiratory complications, particularly in the right lung, even under prophylactic treatment, HSCT is currently in consideration.

In summary, we report a novel mutation in the *NCF2* gene that led to AR-CGD in a young patient with recurrent lung infection even under TMP-SMX and itraconazole prophylaxis combined with IFNγ treatment. Our patient's clinical manifestations illustrate the signs that clinicians must take into account to suspect a diagnosis of CGD even in female children and to trigger diligent immunological and genetic investigations to confirm or discard this hypothesis. Further genetic investigations in the patient's family and her transplanted cousin should be performed for a complete correct genetic counseling.

## Data Availability Statement

This manuscript contains previously unpublished data. The name of the repository and accession number are not available.

## Ethics Statement

This study was approved by the Ethics and Research Committees in Humans of the Sabará Hospital and Institute of Biomedical Sciences, University of São Paulo, in accordance with the Declaration of Helsinki. The patient's parents provided written informed consent before the investigation.

## Author Contributions

JA, EO, and NZ designed, conducted the experiments, and wrote the manuscript. EI conducted the experiments. AL, SC, and FF performed clinical and laboratory data collection and analysis. WA-F performed data analysis, wrote, and reviewed the manuscript. AC-N designed the experiments, wrote, and reviewed the manuscript. AL also reviewed the manuscript.

### Conflict of Interest

JA and EO were employed by Immunogenic Inc. The remaining authors do not declare any commercial or financial relationships that could lead to any potential conflict of interest.

## Abbreviations

CGD: chronic granulomatous disease
TMP-SMX: trimethoprim-sulfamethoxazole
IFNγ: interferon-gamma
NADPH: nicotinamide adenine dinucleotide phosphate
ROS: reactive oxygen species
AR: autosomal recessive
HSCT: hematopoietic stem cell transplantation
BCG: Bacillus Calmette-Guérin.
